# Carers' involvement in decision making about antipsychotic medication: A qualitative study

**DOI:** 10.1111/hex.12616

**Published:** 2017-08-25

**Authors:** Norman J. Stomski, Paul Morrison

**Affiliations:** ^1^ School of Health Professions Murdoch University Murdoch WA Australia

**Keywords:** antipsychotic medication, Carer, decision making, participation

## Abstract

**Background:**

Current Australian mental health policy recommends that carers should be involved in the provision of mental health services. Carers often provide intensive support to mental health consumers and gain detailed insight into their lives. As such, carers could make valuable contributions to well‐informed decisions about mental health consumers' use of antipsychotic medication.

**Objectives:**

The aim of this study was to explore carers' participation in antipsychotic medication decision making.

**Methods:**

Snowball sampling was used to enrol 29 carers in this study. Of these carers, 19 participated in semi‐structured interviews, and ten participated in a focus group. Data were analysed thematically.

**Results:**

Four main themes emerged from the analysis. The findings highlighted that carers typically received little or no information about antipsychotic medication. Carers commonly addressed the shortfall in information by obtaining additional information through online sources or distributing among carer networks material that they had developed themselves. Almost all carers emphasized that they should be involved in decisions about antipsychotic medication, but noted that they were typically excluded. The lack of involvement in medication decisions was a source of frustration, as carers could contribute saliently through sharing detailed knowledge about mental health consumers' lives, address communication gaps that resulted from disjointed care and improve communication between health professionals and mental health consumers.

**Conclusion:**

Health professionals could consider improving the extent to which they collaborate with carers in medication decisions.

## 1 INTRODUCTION

Current Australian mental health policy recommends that carers should be involved in the provision of mental health services. This study explores carers' participation in antipsychotic medication decision making. Snowball sampling was used to enrol 29 carers in this study. Data were analysed thematically. The findings highlighted that carers typically received little or no information about antipsychotic medication. Carers commonly addressed the shortfall in information by obtaining additional information through online sources or carer networks. Almost all carers emphasized that they should be involved in decisions about antipsychotic medication, but noted that they were typically excluded. The lack of involvement in medication decisions was a source of frustration, as carers could contribute saliently through sharing detailed knowledge about mental health consumers' lives, address communication gaps and improve communication between health professionals and consumers.

## 2 BACKGROUND

Carer involvement in service delivery has been recognized as an integral component of mental health care.^1^, ^2^ The increased emphasis on carer participation has to some extent been driven by the shift away from hospitals towards primarily providing mental health treatment in the community, wherein carers are more extensively engaged in supporting consumers.^3^ The development of antipsychotic medication and its subsequent use as the cornerstone of treatment for severe mental illness has been one of the factors behind the increase in delivering mental health care in the community.^4^ Given their involvement in the recovery of mental health consumers, carers may gain important insight into the effect of antipsychotic medication on consumers' lives and make a valuable contribution to well‐informed decisions about the use of medication.

The adverse impact of antipsychotic medication on mental health consumers' lives will also probably be a significant concern for their carers, especially as the side‐effects often impair physical and social functioning, and carers therefore will have an important role in assisting consumers with daily activities.^5^, ^6^, ^7^, ^8^, ^9^ To our knowledge, though, no prior research has explored carers' involvement in decision making about antipsychotic medication specifically. However, other facets of caregiving for mental health consumers have been detailed in several previous studies, which inform the context of this study.

Studies have consistently demonstrated that carers of mental health consumers experience elevated levels of burden and mental distress, and impaired quality of life.^10^, ^11^, ^12^, ^13^ Moreover, recent Australian research reported that almost half of mental health consumer caregivers satisfied criteria for probable psychiatric disorders and were ten times more likely to experience social isolation than other community groups.^3^ Carers often note that mental health services are not meeting consumers' basic needs, which evokes anguish in carers and increases burden.^4^ In addition, carers of mental health consumers commonly report that health professionals do not provide information or advice about the anticipated course of mental illness or mental health care in general, which are also sources of significant distress.^14^, ^15^, ^16^

In summary, the extant literature reports important details about distress and burden among carers of mental health consumers. The literature notably omits studies that explore carers' involvement in decisions made about mental health consumers' antipsychotic medication regimes. It is important to develop an understanding of carer involvement in such decisions as they often have detailed insight into the lives of mental health consumers and could contribute valuable information about the effect of medication. The aim of this study therefore was to explore carers' participation in antipsychotic medication decision making.

## 3 METHODS

A narrative approach was used to guide the conduct of the interviews in this study.^17^ This approach seeks to elicit from the interviewee information about important events and the context in which they occurred. The interviewer aims to exert little influence over the elicited narrative, because the interview's main purpose is to understand social events in terms of the informant's perspective. As such, the narrative approach was well suited for this study, as we sought to authentically reflect the experience of a group of people whose voices have often been marginalized by health professionals and the highly structured care systems in which they work.

### 3.1 Recruitment

Carers of mental health consumers who took antipsychotic medication were enrolled in this study through the use of snowball sampling.^18^ Potential participants were initially identified from the personal contacts of a caregiver who expressed interest in a prior study that we had undertaken. In addition, contact details for potential participants, who had declared interest in sharing their experience, were sourced from an advocacy service. Finally, invitation notices were disseminated through social media and email lists by several carer organizations.

### 3.2 Ethics

The Murdoch University Human Research Ethics Committee granted ethical approval for this study (2015/039). All participants confirmed that they had read the information letter and provided written informed consent prior to the commencement of each interview. Pseudonyms were assigned to all participants to maintain confidentiality.

### 3.3 Data collection

Semi‐structured individual interviews were the principal means through which data were collected, although in two instances, family member pairs were interviewed at the same time, and a single focus group was conducted with a carer support group. All interviews were undertaken by a research assistant, who used recommendations that were developed to promote consumer participation.^19^ The interviews and focus group were audio‐recorded, lasted between 45 and 90 min, and were structured around an interview guide comprising open‐ended questions. Some examples of the initial open‐ended questions are as follows:

1. Tell me about the person you care for?
2. What did the staff tell you about the medication?
3. What information did the staff provide you about potential side effects?

The initial open‐ended questions were followed by probing questions such as:

1. How did you feel about…?
2. How did you respond to…?

At the conclusion of each interview, the following questions were used:

1. Is there anything else you would like to tell me about your experience in caring for…?
2. Is there anything you'd like to tell health professionals, or people thinking about using antipsychotic medication, about your experience in caring for someone using antipsychotic medication?

### 3.4 Data analysis

All interviews and focus group recordings were transcribed verbatim. Thematic analysis was used to analyse the data.^20^ The analysis was mainly focused on delineating broad themes in the textual data sets. An inductive data‐driven process was used to generate the themes, through which the themes were tightly linked to the data and predominantly remained at the semantic level.^20^ All aspects of the data reflected views about the provision of information and involvement in decision making about antipsychotic medication and were coded to explore the manner in which the experience was embodied in each account. The analysis began with line‐by‐line coding, which consisted of considering each line of data on an individual basis, and assigning initial codes that explained small sections of the data.^21^ Next, focussed codes were constructed by combining similar initial codes through the use of constant comparison analysis.^21^ Particularly incisive codes were grouped into preliminary themes and then reiteratively synthesized into overarching themes.

## 4 FINDINGS

The thematic analysis resulted in the identification of four principal themes that provide a rich narrative of carers' participation in antipsychotic medication decision making: Receiving Information about Antipsychotic Medication; Independently Gathering Information about Antipsychotic Medication; Exclusion from Decisions about Antipsychotic Medication; and Contributing to Enhanced Decision Making about Antipsychotic Medication. These themes are detailed in the following sections, and excerpts from the transcripts are provided.

### 4.1 Participant characteristics

In total, 29 carers were enrolled in this study, of which 19 participated in semi‐structured interviews, and 10 participated in a focus group. Most carers were female and typically cared for male mental health consumers. In about three quarters of the cases, carers supported people with a diagnosis of either schizophrenia or schizoaffective disorder.

### 4.2 Receiving information about antipsychotic medication

Participation in decision making depends on the provision, acquisition and understanding of relevant information about medications that enables people to make informed decisions. However, most carers noted that they had been provided with little or no information about antipsychotic medication from the treatment team:

It seems to me to be from looking at his prescriptions and what I paid for his medications, he went onto it in January and I certainly was not told he was put onto it. I was not even told that it had been changed let alone not telling me about the drug.

[Cynthia 165‐167].

Very little. I mean they just say this will fix him or this will help him. When we tried to push them for any more information we were very quickly cut off.

[Paul 97‐98]

Several carers stated that they had received detailed information, but even then there were reservations about the manner in which the information was presented and the stressful context in which it was provided:

There was always consultation with the psychiatrist. When I say always, as much as possible there'd be consultation when we were there about it but the intensity of the information and the intensity of the stress that you are under makes it very, very difficult to really appreciate it. I don't know how much you know about drugs but you start looking at the side effects and possible side effects and it can be from one extreme to another depending on the individual and how they react. So knowing what the potential side effects are doesn't really make any difference. Academically yes, it could happen but it could swing so widely so you can't predict it.

[Tom 124‐131]

As the above excerpt demonstrates, information about antipsychotic medication could be presented to carers in more concise terms, which perhaps emphasizes the most likely effects over the full spectrum of effects. In a similar vein, some carers noted that medication information should highlight how carers would need to respond to the impact of antipsychotic medication on consumers' lives:

If you are delivering information, you can't just hand the carers and family same damn information you hand the client. You might feel tired – great! So for the carer it might be – This person [consumer] might feel tired which will put more responsibility on you to make sure that they take their medication at the right point. If you are taking them home, who is responsible for that? And so the carers don't actually know crap. And that then forces there to be a discussion with the clinicians about, do they get a nurse to come in to remind them? Because otherwise they are expecting the family to and if they aren't aware of that, they will just say yes to get their family member [consumer] out [of hospital].

[Kisia 405‐413]

Moreover, developing an understanding of the manner in which carers will need to support mental consumers also depends on realistic details being made available to carers about the effectiveness of antipsychotic medication:

I think before a person is going to be taking antipsychotics, I think the family has to be taken outside and given the statistical information about antipsychotics. I don't think you need to kill hope, I think we should always have hope in this life. But I think it's such a serious thing that we need to be realistic on what we have.

[Irene 372‐375]

### 4.3 Independently gathering information about antipsychotic medication

The failure to provide information to carers or the unsuitability of the information that was provided prompted most carers to independently source information about antipsychotic medication and mental illness. This initiative was necessary to compensate for the shortfall in appropriate information provided by health professionals. In a minority of the cases, carers did not actively seek out information because the medication was effective:

I have done a bit of reading and I read all the blurbs that are attached to the medications when they arrive. But I must admit these days I don't, I feel a little bit out of touch but because it all seems to be working I just left it alone really.

[Belinda 201‐203].

The carers, more typically though, took the initiative in obtaining information, often after observing deterioration in the condition of the consumer they cared for:

We could really hardly get [the consumer] back to his seat, his legs were just giving way. And this was so unusual and a couple of other people who of course knew him because he worked there, they came to help and we managed to get him back to the first pew we came to, not our seat. That made me very suspicious what was happening with [the consumer].

[Jill, 125‐129]

Some of the details about medication and mental illness were sourced from books, but it was more common for carers to access information from the online sites: “Since the advent of the internet it's been a lot easier because a lot more information has become available” [Em 182]. Acquiring knowledge about the medication was important as it enabled some form of participation in decision making with health professionals:

You really need to be involved with the dosage and what the medications they are using. Find out what it's all about, get some information. Go and Google it, whatever. You've got to have the information and to be able to talk to the doctor.

[Paul 429‐432]

Finally, some carers drew on their own experience to develop information resources that could be shared with other carers:

We teach carers strategies, how to be assertive, the medical words to use in mental health, so when you go there, to that professional, you know what to say to stay calm, to be assertive, don't take no for an answer, make them listen to you. That's how I became …because I had to. So that's all the strategies in our Well Ways program that we teach carers. So when they finally get there, it's so hard but use all those strategies, speak to a professional in a … use the medical words that go with mental illness. So I think if families can be taught all those strategies in dealing with professionals, they are going to be miles ahead by the time they get there. But the trouble is by the time they get there, they haven't done any of this education, it's only later they do any education.

[Cathy 332‐339]

### 4.4 Exclusion from decisions about antipsychotic medication

Most carers emphasized that they should be involved in medication decisions but that they were also typically excluded from decision making: “I have not been involved in anything to do with his medication with any psychiatrists because they won't talk to me” [Angela 259‐260]. The experience of exclusion was a source of frustration for carers and sometimes evoked anger:

There were times when I would be just completely unapproachable because I would be angry and I carried it over into our personal relationship rather than leaving it at the door with the doctors or whatever because I really did want answers about why this was happening, or why they wanted to take so many blood tests, what it meant to have your white cell count go up and all that sort of thing.

[Em 48‐52]

Moreover, the lack of collaboration was especially galling for several carers as they had detailed knowledge of the consumers' lives that could be used to inform medical decisions:

I think you feel quite a sense of injustice about that because if you've observed someone close range day‐by‐day for a long time then you do have insights which others don't, couldn't possibly have… So I guess that's a hard part of the experience that you feel locked out of decisions, locked out of you know of the insights you have on the effects of medication and changes of medication and what the outcomes are and asking questions about dosage.

[John 163‐171]

Some carers indicated that there was genuine collaboration in decision making, and it seems clear that the facilitation of communication around the effects of medication would have been beneficial for the consumers more generally:

We were in contact with the psychiatrist about our observations about whether it seemed to be agreeing with him or not. Watching for side effects. We had a really good partnership really, all three of us I think. Us as parents, she as the doctor, and him. It worked very well

[Belinda 68‐72]

To reiterate, though, most carers were generally excluded from decision making about antipsychotic medication. Confidentiality issues were commonly cited as a reason for not involving carers in these decisions: “usually what people say to you is well we can't talk to you about this because it's confidential between us and the client” [John 172‐173]. Nonetheless, while carers recognized the importance of maintaining the mental health consumers privacy, some felt that health professionals could be more accommodating and explore ways in which they could communicate more openly without breaching confidentiality:

There's always the confidentiality barrier that many, many clinicians use because they can't see the wood for the trees either. They can still be empathetic with the family, they could say “I can't discuss that with you but let's see what we can talk about.

[Cynthia 220‐223].

It seems likely that issues around the protection of confidentiality present as a complex dilemma for health professionals. On the one hand, maintaining mental health consumer privacy should be a paramount concern. Yet consumers in the community may depend on the support of carers to stay well, and carers may be unable to adequately fulfil that caring role unless they have a clear understanding of the consumer's needs and the treatment and care plan:

They would send him home to me, they didn't even often tell me when they were sending him home. If they are sending our family members home to us, we have a right to know what's wrong with them and what they are taking and what we should look for especially with some of the antipsychotics.

[Amanda 25‐30].

The rationale for not informing carers when consumers are discharged from mental health facilities is not clear. The failure to notify carers about the discharge of consumers may have resulted from a genuine concern about confidentiality, but in some cases, a breakdown in communication was clearly responsible:

I got a call on Friday at work saying “We need to talk about a discharge plan. I am going on leave but I will get someone to follow up with you next week just to make sure everything is sorted.” She wasn't happy with the discharge. Now my mums' electricity had been cut off. She didn't trust the hospital so I had her keys, I had her phone, I had her ID, I had everything. I had her money; she had nothing. Called back but they were shut at that point. And then I get a call just before 8 o'clock the next morning on my way to work so I don't answer it. So I get to work and give them a call. They put her on a bus up to Geraldton, with no ID or nothing. They were supposed to call me before they did it but the doctor said no, she was ready to leave. That doesn't mean leave for the 7.50 bus in the morning!

[Kisia 244‐252]

Finally, although carers were typically excluded from decision making about antipsychotic medication, they were able in some instances to overcome the sense of powerlessness as a carer and influence decisions through concerted effort and practice as an advocate:

You really always have to push yourself in that regard and if you're not confident then you wouldn't. I've had contact with a lot of carers over the years and many of them just feel powerless in that situation and feel unable to put their case and are easily dismissed in terms of any significant input that they may have to make. But in my case it's different because I became an advocate and I dealt at high levels in advocacy so it makes a difference.

[John 192‐197]

In other cases though, carers were only able to influence medication decisions after gaining the support of advocates (from a formal advocacy service) who attended meetings between the carers and health professionals:

[the advocate] said “This is going to take a lot of time to get him off this but [the carer] obviously would like him to come off, can you see that that happens?” So we planned to see [the psychiatrist] a week later and when we saw her a week later, [the advocate] asked “How far off we had come from getting [the consumer] off this medication?' and [the psychiatrist] said “Well he is off

[Jill 211‐216]

### 4.5 Contributing to enhanced decision making about antipsychotic medication

Several carers noted that they could improve decision making through providing information that consumers may be unable to disclose perhaps because they do not wish to or because they are unwell:

They [carers] really need to be included in decisions when the person is unwell, even if they are an adult. You really need collateral information from carers because you know, they [consumers] are not always going to tell you [professionals] what is happening. Sometimes they can't tell you so I'd like to have a less of them and us kind of perspective and a bit more of a partnership role I think.

[Belinda 322‐327]

Carers also noted how they could enhance medical decisions through bridging communication gaps that resulted from disjointed care. These gaps emerged from frequent changes in mental health staff, inefficiencies in record keeping or impaired communication between health professionals:

It's almost as if every 2 weeks you have to tell the story about [the consumer]. It's just totally incomprehensible. Maybe they have too many notes for him or too many files for him. We don't have a system yet that updates things very quickly in the computer. So it seems to me I spend my time telling people the same story all the time. Every 2 or 3 weeks. Also the coordination between doctors and nurses and welfare workers is not there.

[Irene 48‐53]

The manner in which medication‐related information was provided to mental health consumers was also an aspect of communication that carers felt could be improved. In particular, carers voiced concern that health professionals did not clearly communicate the most typical medication effects and that carers could assist in clarifying these issues for consumers:

I just don't find that a lot of mental health professionals are very mindful of how some of the information they are giving is received by someone who has a mental impairment. Or understood, you know…For someone who is severely psychotic, who goes to their meeting with their doctor who says “Well we can try you on this tablet, these are the side effects.” And they go “Oh mum, it does this and it does that.” Because somewhere the consumer has not comprehended that these are just the possible side effects, that it doesn't mean that it will happen to you. But for someone living with paranoid schizophrenia, they are going to internalize that and start getting freaked out about it… they don't transfer in the real practical sense of what they mean. So if I am not at that appointment with my son and he comes home and he says “Well the doctor wanted to try me on this tablet. This is what it does, I'm not bloody taking that.

[Jane 217‐241]

## 5 DISCUSSION

The carers in this study typically reported that they had received little or no information about antipsychotic medication. Even when such information had been provided, carers expressed reservations about the content of the material. Carers commonly addressed the shortfall in information by obtaining additional information through online sources or distributing among carer networks material that they had developed themselves. Almost all carers emphasized that they should be involved in decisions about antipsychotic medication, but noted that they were typically excluded from these decisions. The lack of involvement in medication decisions was a source of frustration, as carers could contribute valuably through sharing detailed knowledge about mental health consumers' lives, address communication gaps that resulted from disjointed care and improve communication between health professionals and mental health consumers.

Mental health consumers and health professionals typically express interest in collaborative decision making.^22^, ^23^ Yet health professionals commonly state that they do not include mental health consumers in decisions because of a perceived lack of capacity.^22^, ^23^ The findings of the present study demonstrate that exclusion from decision making also extends to carers of mental health consumers, who have the right to be involved in decision making and clearly have the capacity to make worthwhile contributions.^1^, ^2^ The exclusion of carers and consumers suggests that the prevailing culture in mental health services may be controlling, certainly in relation to the experiences of the participants in this location. Hence, mental health services may need to reassess the extent to which consumers, and especially carers, could be more effectively included in decision making with respect to the prescription, delivery and evaluation of antipsychotic medication.

The importance that carers placed on being involved in decision making about antipsychotic medication mirrors the call in the most recent National Mental Plan for enhanced participation of carers in the provision of mental health services.^2^ Recommendations alone, however, are not adequate and formal structures and processes may need to be implemented to ensure that the level of carer engagement is appropriate for the individual needs of both consumers and carers. One such framework is the Family Involvement in Interpersonal Health Care Processes model.^24^ This framework articulates a detailed series of pathways, which can be summarized across three main interpersonal areas: relationship rapport, information exchange and medical decision making. Relationship rapport encompasses empathy, trust, respect and other similar interpersonal processes, which result in a genuine collaborative alliance between health professionals, consumers and carers.^24^, ^25^ Information exchange depends on health professionals receiving information that enables them to make an accurate diagnosis and deliver appropriate care.^24^, ^26^ It also relies on the provision of information that allows consumers and carers to understand the illness and care options, benefits, adverse affects and uncertainty about the results of treatment. Finally, medical decision making involves an iterative process that unfolds over time in consultation with consumers and caregivers.^24^, ^27^ In essence, health‐care issues and options are communicated in clearly understandable terms that enable carers, and especially consumers, to evaluate them in the light of their own experiences, preferences and values.

While the absence of appropriate information about antipsychotic medication was notable, in those instances where information was provided, carers highlighted a number of salient points about that information. It was noted, for example, that the range of side‐effects detailed was so extensive that it was difficult to determine which side‐effects were most likely to occur. The large volume of information also tended to result in consumers' catastrophizing about the detrimental adverse effects, although it should be noted such an attitude is not entirely unwarranted as antipsychotic medication commonly produces severe side‐effects. In addition to highlighting the most likely adverse effects, information should present the likely benefits of antipsychotic medication, which are often marginal.^28^ Finally, it is incumbent of health professionals to provide information about the potential impact of antipsychotic medication on consumers' lives and how this might influence the type of support carers would need to provide to consumers, sometimes in an on‐going way when medication is recommended for long periods of time. In summary, it would be worthwhile to conduct further studies to develop and workshop information that presents the most likely beneficial and adverse affects of antipsychotic medication, and the types of strategies needed to support mental health consumers as they deal with the consequences of these medications.

In some instances, mental health consumers may have concerns about the extent to which their families are involved in mental health‐care decision making. These concerns, though, can usually be accommodated through using communication processes that enable respectful family participation.^29^ This approach to fostering family involvement is based on the use of release forms that specifically detail information that health professionals can share with families.^30^ It may be the case that some mental health consumers do not want health professionals to provide any information to their families. However, health professionals are only obliged to not disclose confidential information and can consider what types of information could be given to families without breaching confidentiality. Such non‐confidential material can include basic information about mental illness, which may include aetiology, general prognoses, warning signs and symptoms, and standard treatment plans.^30^

## 6 LIMITATIONS

The findings presented here reflect the experience of a moderate number of caregivers, who reside in a single Australian state. Also, the sample was self‐selected and may have had a particular interest in the researched area. Our results therefore should be viewed carefully as the material might not be representative of carers' perspectives in general. Nonetheless, the findings of this study provide a rich, novel description of diverse carer views in regard to the use of antipsychotic medication, which may be of benefit to health professionals in informing the delivery of mental health care.

## 7 CONCLUSION

This study builds an understanding of the carers' role when supporting mental health consumers who take antipsychotic medication; their concerns about information and decision making, the sense of exclusion and missed opportunity to contribute usefully to the care process in a collaborative way. Overall, the findings demonstrate the health professionals need to substantially improve the extent to which they collaborate with carers in medication decisions, especially as current Australian mental health policy mandates the involvement of carers in the delivery of services to mental health consumers. Carers often provide essential, intensive support to mental health consumers during recovery, and therefore, their views and experiences should be an integral component of the assessment and production of treatment and health‐care services. Further, gaining a more detailed appreciation about carers' lives will assist health professionals to develop appropriate approaches to support carers as they walk alongside mental health consumers in the recovery journey.
